# A web-based group course intervention for 15-25-year-olds whose parents have substance use problems or mental illness: study protocol for a randomized controlled trial

**DOI:** 10.1186/s12889-016-3691-8

**Published:** 2016-09-23

**Authors:** Tobias H. Elgán, Nicklas Kartengren, Anna K. Strandberg, Maria Ingemarson, Helena Hansson, Ulla Zetterlind, Johanna Gripenberg

**Affiliations:** 1 STAD, Centre for Psychiatry Research, Department of Clinical Neuroscience, Karolinska Institutet & Stockholm Health Care Services, Stockholm County Council, Stockholm, Sweden; 2 School of Social Work, Faculty of Social Sciences, Lund University, Lund, Sweden; 3 Clinical Health Promotion Centre, Department of Health Sciences, Lund University and Skåne University Hospital MAS, Lund, Sweden

**Keywords:** Child of impaired parents, Children of alcoholics, Mental illness, RCT, Web-based intervention, Digital intervention, Internet, Social media, Chat, Group

## Abstract

**Background:**

Depending on the definitions used, between 5 and 20 % of all Swedish children grow up with at least one parent suffering from alcohol problems, while 6 % have at least one parent who has received inpatient psychiatric care, conditions that may affect the children negatively. Nine out of ten Swedish municipalities therefore provide support resources, but less than 2 % of these children are reached by such support. Delivering intervention programs via the Internet is a promising strategy. However, web-based programs targeting this at-risk group of children are scarce. We have previously developed a 1.5-h-long web-based self-help program, *Alcohol & Coping*, which appears to be effective with regards to adolescents’ own alcohol consumption. However, there is a need for a more intense program, and therefore we adapted *Kopstoring*, a comprehensive Dutch web-based psycho-educative prevention program, to fit the Swedish context. The purpose of the program, which in Swedish has been called *Grubbel*, is to strengthen protective factors, such as coping skills and psychological well-being, prevent the development of psychological disorders, and reduce alcohol consumption.

**Methods/design:**

The aim of the current study is to evaluate the effectiveness of *Grubbel*, which targets 15–25-year-olds whose parents have substance use problems and/or mental illness. Specific research questions relate to the participants’ own coping strategies, mental health status and substance use. The study was initiated in the spring of 2016 and uses a two-armed RCT design. Participants will be recruited via social media and also through existing agencies that provide support to this target group. The assessment will consist of a baseline measurement (t0) and three follow-ups after six (t1), 12 (t2), and 24 months (t3). Measures include YSR, CES-DC, Ladder of Life, Brief COPE, AUDIT-C, and WHOQOL-BREF.

**Discussion:**

Studies have revealed that the majority of children whose parents have substance use or mental health problems are not reached by the existing support. Thus, there is an urgent need to develop, implement, and evaluate novel intervention programs and disseminate successful programs to a broader audience. This study, investigating the effects of a web-based intervention, therefore makes an important contribution to this field of research.

**Trial registration:**

ISRCTN10099247. Retrospectively registered on August 31, 2016.

## Background

Children growing up in families with parental substance use problems or mental illness are at risk for a number of psychological problems (reviewed in e.g., [1–3]), including depression and anxiety disorders, as well as behavioural problems [3–5]. Furthermore, these children may also experience poor intellectual, cognitive, and academic achievement [6, 7], domestic physical abuse [8], and are at risk for earlier drinking onset [9] and for developing substance use problems themselves [10–12]. Children affected by parental substance use problems or mental illness therefore comprise a target group for selective intervention and prevention strategies (reviewed in e.g., [13, 14]).

International estimates have revealed that the problem is widespread, and the proportion of children with at least one parent with substance use problems ranges from about 8 to 30 % [13, 15–18], while about 12–39 % have at least one parent with a mental health problem [19–22]. Reasons for the variation in the figures may include how the problem is defined, the methodology of data collection, and cultural differences. In line with this, Swedish estimates have revealed that approximately 5 % of all children have at least one parent with a substance use disorder [23], while 17–20 % grow up in families where at least one parent has risky alcohol consumption or substance use problems [24–26]. Moreover, about 6 % of all Swedish children have at least one parent who has received inpatient psychiatric care [25].

The Swedish municipalities alone account for the vast majority of support offered to children whose parents have substance use problems or mental illness. In fact, during 2015, more than 90 % of all municipalities provided support resources or referred children of substance-abusing parents to adequate support [27]. The corresponding number concerning support for children whose parents have mental illness was 73 % in 2009 [28]. However, the vast majority of children affected by parental problems are not reached by the existing support, and estimates reveal that not even 2 % of all children growing up with parental substance use problems receive support. Thus, there is a tremendous gap between those who might be in need of support and those who actually receive it, and reasons to this gap may be attributed to problems with accessibility of intervention programs and difficulties in identifying and attracting children into these programs [13, 24].

Digital interventions delivered via for instance the Internet or mobile phone applications provide one promising way to reach out and support a larger number of individuals (reviewed in [29–31]). With regards to adolescents and young adults, this seems attractive since they generally have great experience with digital technology and social media. Furthermore, research has shown that adolescents regard the Internet as particularly appealing since it provides an accessible and anonymous way of seeking help [32]. Web-based interventions targeting a wide range of conditions have also been found to be effective, which was concluded in a comprehensive review covering 92 studies including a total of 9764 participants, where the mean effect size was found to be similar to that of traditional “face-to-face” therapy [33]. It should be noted that the vast majority of digital interventions reported in the literature have been designed for and tested on adults. However, although the number of web-based interventions targeting children or adolescents is increasing [34–40], the number of interventions aimed at children whose parents have substance use problems or mental illness is still scarce [13, 41–43].

We have previously developed a web-based therapist-guided self-help program called *Alcohol & Coping*, which targets 15–19-year-olds with at least one parent with alcohol problems. The intervention has been evaluated in a randomized controlled trial (RCT), which revealed that about 40 % of the adolescents in the study had self-reported risky alcohol consumption at the baseline assessment (personal communications THE, HH, UZ). Results further revealed that the intervention appeared to be effective with regards to adolescents’ own alcohol consumption. However, *Alcohol & Coping* is a relatively brief intervention (about 1.5 h) and there is a need for a more intense program. We have therefore translated and culturally adapted a comprehensive Dutch web-based group prevention program called *Kopstoring* into a Swedish context. *Kopstoring* is based on psycho-educative principles, and consists of eight weekly online chat group meetings, each lasting for about 1.5 h, and an additional ninth follow-up meeting. Each meeting focuses on a particular theme and is moderated by one or two trained professionals. The *Kopstoring* program is currently being evaluated in the Netherlands [42, 44]. The present text reports on the design of a study investigating the effectiveness of the Swedish version of *Kopstoring*, called *Grubbel*.

### Objective and research questions

The main objective of this study is to evaluate the effectiveness of the group intervention *Grubbel* targeting 15–25-year-olds with at least one parent with a substance use problem or mental illness. We hypothesize that in comparison with a control group who receives care as usual (CAU), participants in the intervention group will show improved coping skills, reduced symptoms of depression and behavioural problems, improved quality of life, and a reduction in alcohol consumption.

Another objective is to study the intervention implementation as regards the intervention’s content and structure and its feasibility in use. What has been successful in the interaction between participants and the moderators, which were the facilitating and hindering factors of implementation, and what can be said about the applicability of the technical intervention platform?

## Methods/design

**Fig. 1:**
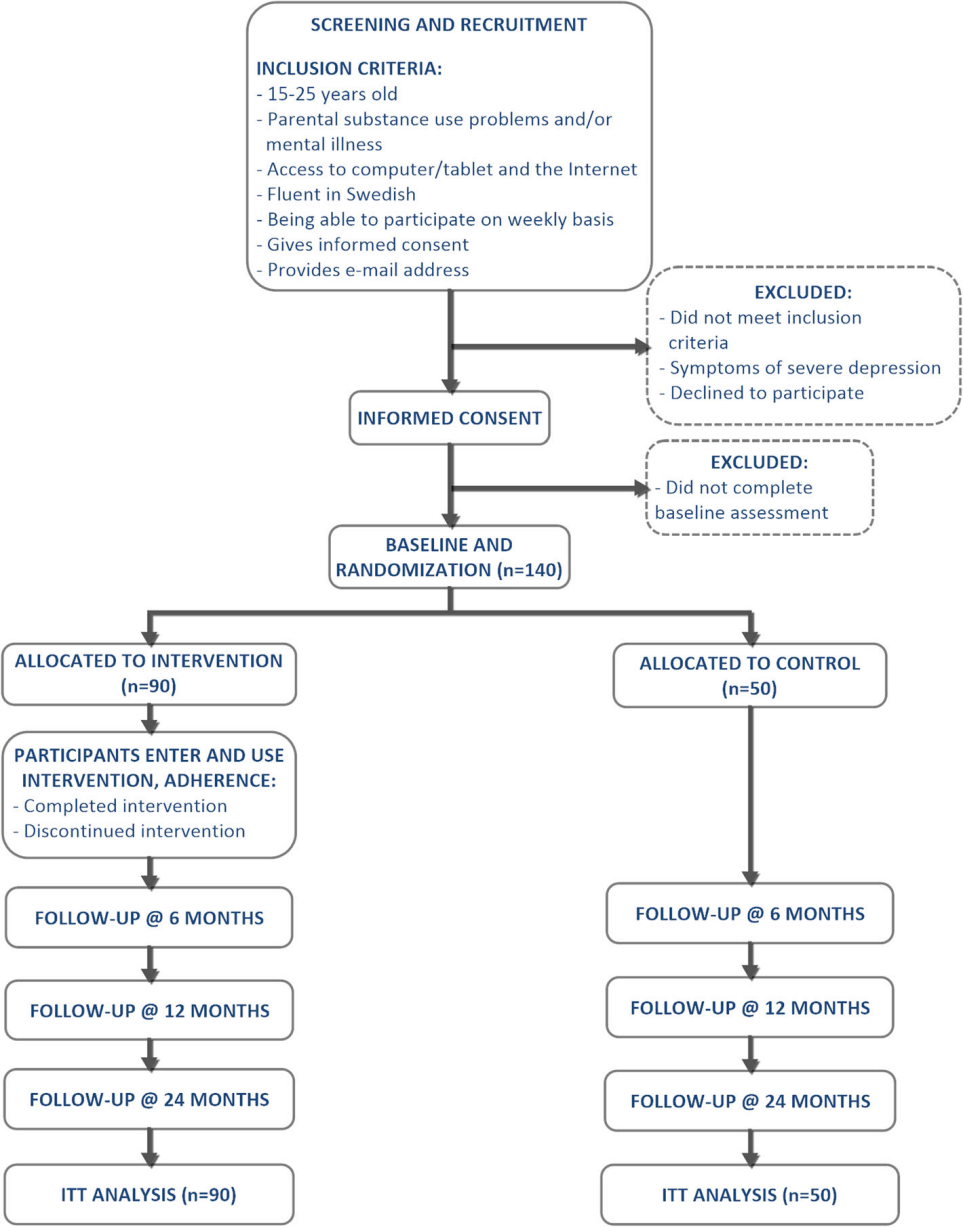
Flow-chart diagram depicting the design of the study. The participants are unequally allocated (ratio 1.8) in favour of the intervention group

The study was initiated in April 2016 and consists of a quantitative two-armed RCT (Fig. 1) and a qualitative implementation study.

### The target sample

The aim is to include at least 140 adolescents (aged 15–25 years) who perceive at least one parent to have a substance use problem and/or mental illness. Additional inclusion criteria (Fig. 1) are as follows: having access to a device, such as a computer or tablet with internet access, being fluent in Swedish, and having consented to participate. Participants who reveal symptoms of severe depression or show indications of either suicidal or self-injurious behaviour will be excluded from the study and referred to appropriate care.

### Recruitment and screening

Participants will be recruited primarily via social media (i.e., Facebook and Instagram) and also through existing agencies, authorities, and organisations such as health care centres, social services, non-profit organizations, and self-help groups aimed at adult children of alcoholics, which all have in common that they provide support to this target group.

Those who are interested in participating will be referred to the study webpage (www.grubbel.nu), which contains further information about the study and a link to an online screening survey. In addition to demographic questions such as age and gender, the participants will be screened for having parents with alcohol problems through the Children of Alcoholics Screening Test (CAST-6). The CAST-6 is a six-item measure originally designed to assess whether or not individuals perceive their parents’ alcohol consumption as problematic, and has been proven to be a useful screening instrument which compares well with other measures [45, 46]. The CAST-6 is reliable (*r* = 0.94), has high internal consistency (*r* > 0.92), and is valid (*r* = 0.93) as compared with the original 30-item version using the recommended cut-off score of 3 or higher [45, 46]. The participants will also be asked to respond to the question: “Do you perceive any of your parents to have a mental illness?”.

Those who have 3 or more points on the CAST-6 or answer yes to the question on parental mental illness will be asked to answer questions concerning their motivation and possibility to participate in the group course on a weekly basis, and also to fill out the CES-DC questionnaire, which measures symptoms of depression (see below). Those who have at least one parent with a substance use problem or mental illness, who are motivated and have the opportunity to participate in the group course on a weekly basis, and do not have symptoms of severe depression as measured by the CES-DC, will be asked to consent to study participation and provide an e-mail address.

### Assessment

The assessments for this study will consist of a baseline measurement (t0), which will take place before randomization, and three follow-up measurements after six (t1), 12 (t2), and 24 months (t3). Participants will be invited to each assessment by e-mail, and if necessary up to three reminders will be sent at five, ten, and 15 days after the first invitation, respectively. All assessments will be in the form of an online survey distributed via the survey data collection tool Easyresearch (QuestBack, Norway). In order to increase the response rate, participants will be offered an incentive of one cinema gift certificate, corresponding to approximately 11 Euros, for each assessment. The resulting survey contains 216 questions and takes approximately 30 min to complete.

### Outcome measurements

#### Primary outcomes

To measure depressive symptoms during the preceding week, the Center for Epidemiological Studies Depression Scale for Children (CES-DC) will be used [47]. This scale consists of 20 items with response options on a four-point Likert scale. It measures depressive symptoms during the preceding week and is a valid measure of general childhood psychopathology [48]. The scale has shown high internal consistency (α = 0.91) and has been proven to be valid among Swedish adolescents when compared with the Beck’s Depression Inventory (*r* = 0.81) [49].

Participants’ coping strategies will be measured using the Brief COPE, which consists of 28 items using four-point Likert scales and has been proven to have acceptable high internal consistency (α = 0.58–0.92) [50, 51].

To measure quality of life, the World Health Organization’s Quality of Life Questionnaire (WHOQOL-BREF) will be used. The scale contains 26 items using five-point Likert scales and measures quality of life in four principal domains: physical health, mental health, social relations, and environment [52]. This instrument has demonstrated good to excellent psychometric properties with Cronbachs’s α ranging from 0.68 to 0.82, and construct validity (item-total correlations) with r ranging from 0.45 to 0.70 [53].

Alcohol consumption will be assessed using the short version of the Alcohol Use Disorders Identification Test (AUDIT-C) [54], assessing frequency of drinking, quantity consumed, and frequency of heavy episodic drinking (i.e., binge drinking). The instrument has shown satisfactory internal consistency (α = 0.80) and good validity (AUROC = 0.85) [55].

#### Secondary outcomes

Overall life satisfaction will be measured using the Ladder of Life, asking about each participant’s past, present, and future rating of his/her life. The original version was intended for adults and asks the respondents to reflect on their life over a 5-year perspective [56]. A modified version for children and adolescents, using a shorter timeframe of 1 year, will be used in this study [57].

To measure the participants’ competencies and behavioural problems, the Youth Self-Report (YSR) questionnaire will be used. It consists of 119 problem behaviour items that form internalizing and externalizing scales, and has been demonstrated to be valid and to have good internal consistency [58, 59].

#### Qualitative interviews

Focus group interviews will be conducted with the trained professionals who moderate the intervention program during the study. The interviews will be based on interview guides containing questions regarding for instance the intervention content and structure, and its feasibility in use. What has been successful in the interaction between the participants and the moderators? How well does the technical intervention platform work? What were the facilitating and hindering factors of implementing the intervention?

### Allocation/Randomization

Participants will be randomly allocated to one of the two study groups after completing the baseline assessment. An unrestricted random allocation sequence will be generated by an external researcher using the Random Allocation Software [60]. Participants will be informed about their allocation by e-mail. Those participants who are randomized to the intervention group will receive further information about the study and about being assigned to a *Grubbel* group. The participants can choose between different dates for the group course, so they start on a day of their preference. Information about how to access the group course will be sent to the participants by e-mail. All participants will receive information about other support and information available according to care as usual (CAU), as described below.

### The intervention

The program *Grubbel* is a manual-based and well-structured prevention program and consists of eight consecutive weekly online chat-meetings, each lasting 1.5 h, and a ninth follow-up meeting one week after the last meeting. The aim of *Grubbel* is to strengthen protective factors such as coping strategies and psychological well-being, to decrease alcohol consumption, and to prevent the development of psychological disorders. The meetings take place in an online group chat tool (Campfire, Basecamp, IL, USA). The group chat is password-protected and encrypted using SSL (Secure Sockets Layer) technology and the same 4–8 participants in each group meet one another online during each session. Each meeting is moderated by one or two trained professionals (prevention and social workers, and psychologists from mental health and addiction centres), who have previous experience from working with the target group, and have participated in a two-day *Grubbel* course held by our research group.

**Fig. 2:**
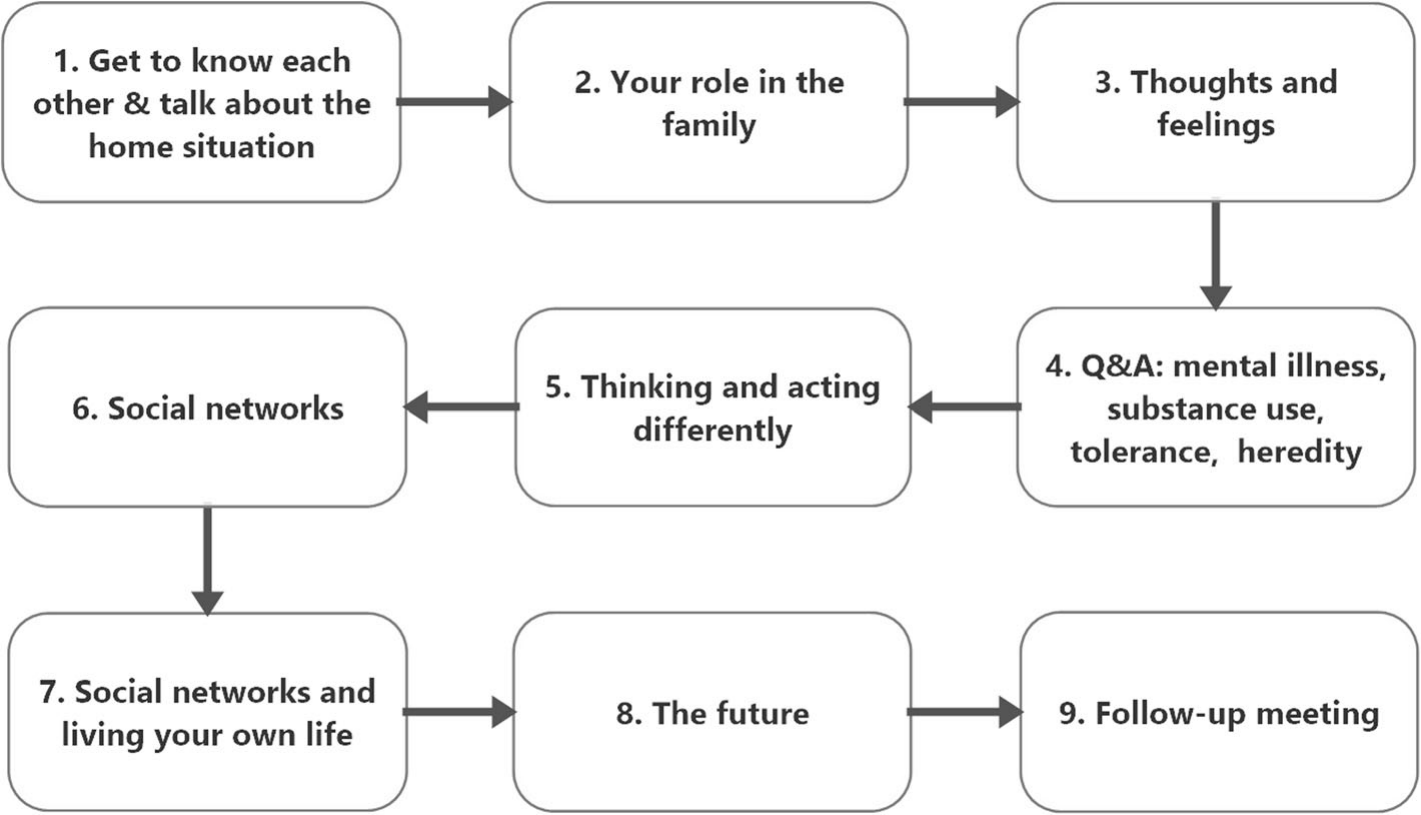
Themes of the program sessions. Each session lasts for about 1.5 h and participants receive a homework assignment which is to be discussed at the subsequent session

Each session focuses on a particular theme (Fig. 2) and the themes are: (1) getting acquainted with the home situation, (2) roles in the family, (3) thoughts and feelings, (4) questions and answers about mental health problems, (5) different behaviour patterns, (6) social networks, (7) leading your own life with regards to social networks, and (8) the future. The ninth session is a follow-up session. After each session, the participants are required to complete a homework assignment to be discussed at the subsequent session. Previous research conducted by our research group has revealed that adolescents whose parents suffer from alcohol problems have a high level of risky alcohol consumption (personal communications THE, HH, UZ). An addition to the original *Kopstoring* manual was therefore made, with the objective of providing structured information about risky use of substances, addiction, and heredity. The fourth theme was also modified to include questions and answers about risky alcohol use, addiction, and heredity (Fig. 2).

### The control

The control condition consists of unrestricted access to CAU. In Sweden, the majority of support to the target group is provided or coordinated via municipalities’ social services, and includes for instance support group activities or individual counselling. In severe cases, children may be referred to the Child and Adolescent Psychiatric Services. Support may also be provided via school health care services and non-profit organizations. In addition, there are a few websites that contain specific information and support for our target group.

### Sample size

The sample size calculation is based on the ability to detect a medium or larger effect size, corresponding to a standardized mean difference (Cohen’s d) of > 0.05 [61]. Based on differences between means, an *a priori* calculation of the estimated sample size, using the G*Power software [62], and accounting for an unequal allocation ratio of 1.8 in favour of the intervention group, reveals that 140 participants need to be included in the trial, 90 in the intervention group and 50 in the control group (power = 0.80, α = 0.05, 2-tailed).

### Analyses

#### The effectiveness study

Data will be analysed according to the intention-to-treat (ITT) principle, accounting for all included participants regardless of whether or not they have responded to the follow-up assessments. Missing data will be handled using the Missing Value Analysis routine in the SPSS software (IBM SPSS Statistics 23, IBM Corporation, NY, USA).

Data analyses will consist of comparing outcome measurements at baseline (t0) and subsequent follow-up (t1, t2, and t3) assessments, with regards to within-group and between-group comparisons.

#### The implementation study

The qualitative data will be analysed with thematic analysis in order to discover the main themes connected to the study aim and research questions.

## Discussion

This protocol describes the study of a Swedish version of the Dutch prevention program *Kopstoring*, called *Grubbel*, which is a web-based prevention program targeting 15–25-year-olds whose parents have substance use problems and/or mental illness. An addition to the original *Kopstoring* manual has been the inclusion of a more structured means of providing information relating to risky use of substances, addiction, and heredity. The effectiveness of *Grubbel* will be evaluated in a two-armed RCT, where the intervention group will be compared with CAU.

### Strengths and limitations

This study has a number of strengths. First, *Grubbel* is based on the Dutch *Kopstoring* program. *Kopstoring* is currently being evaluated in the Netherlands [42, 44], but results regarding the effects of the program have not yet been published. Nonetheless, this study will not only add to the evidence of the *Kopstoring* program, but also to web-based interventions in general and, in particular, to the relatively unexplored field of interventions targeted at adolescents whose parents have substance use problems and/or mental illness. Furthermore, there has been a request among Swedish professionals to develop a web-based intervention program for children and adolescents growing up in families with substance use problems, which increases the chance of widespread dissemination of the intervention if it proves to be effective. Finally, this study combines an RCT study, considered to be the strongest experimental design with regards to participant allocation bias, with a qualitative study of the intervention implementation.

There are also limitations to this study. One limitation concerns selection bias and thereby external validity, as recruitment requires either that individuals click on our Facebook ads to be considered for inclusion, or that they have been in contact with existing support. Thus, our sample may have certain personality traits, such as a help-seeking personality, possibly not seen in a broader audience. Furthermore, *Grubbel* is a relatively intense program and some individuals may not be included due to their inability to allocate 1.5 h per week to participate in the group chat. Among those who are included in the study, this may also pose a problem, and we may experience a lower degree of adherence.

### Implications for practice

Studies have revealed that the majority of children whose parents have substance use or mental health problems do not receiving existing support. In addition to problems with identifying individuals and recruiting them to available intervention programs, a related issue is how to make face-to-face intervention available in rural areas. This has been an issue raised among professionals working in the rural northern part of Sweden and a web-based solution has been requested. Thus, there is an urgent need to develop and evaluate novel intervention programs, such as web-based interventions, and disseminate successful programs to a broader audience. If this study demonstrates sufficient effectiveness in *Grubbel*, our aim is to implement and disseminate the intervention throughout Sweden, which will be a cost-effective means to provide support. This study therefore makes an important contribution, not only to this field of research but also to agencies, authorities, and organisations that provide support to this target group.

## References

[1] Johnson JL, Leff M (1999). Children of substance abusers: overview of research findings. Pediatrics.

[2] Lieberman DZ (2000). Children of alcoholics: an update. Curr Opin Pediatr.

[3] Sher KJ (1997). Psychological characteristics of children of alcoholics. Alcohol Health Res World.

[4] Ohannessian CM, Hesselbrock VM, Kramer J, Kuperman S, Bucholz KK, Schuckit MA, Nurnberger JI (2004). The relationship between parental alcoholism and adolescent psychopathology: a systematic examination of parental comorbid psychopathology. J Abnorm Child Psychol.

[5] Spieker SJ, Larson NC, Lewis SM, Keller TE, Gilchrist L (1999). Developmental trajectories of disruptive behavior problems in preschool children of adolescent mothers. Child Dev.

[6] Casas-Gil MJ, Navarro-Guzman JI (2002). School characteristics among children of alcoholic parents. Psychol Rep.

[7] McGrath CE, Watson AL, Chassin L (1999). Academic achievement in adolescent children of alcoholics. J Stud Alcohol.

[8] Velleman R, Templeton L, Reuber D, Klein M, Moesgen D (2008). Domestic abuse experienced by young people living in families with alcohol problems: results from a cross-European study. Child Abuse Rev.

[9] Rothman EF, Edwards EM, Heeren T, Hingson RW (2008). Adverse childhood experiences predict earlier age of drinking onset: results from a representative US sample of current or former drinkers. Pediatrics.

[10] Anda RF, Whitfield CL, Felitti VJ, Chapman D, Edwards VJ, Dube SR, Williamson DF (2002). Adverse, childhood experiences, alcoholic parents, an later risk of alcoholism and depression. Psychiatr Serv.

[11] Chassin L, Rogosch F, Barrera M (1991). Substance use and symptomatology among adolescent children of alcoholics. J Abnorm Psychol.

[12] Windle M (1997). Concepts and issues in COA research. Alcohol Health Res World.

[13] Cuijpers P (2005). Prevention programmes for children of problem drinkers: a review. Drug Educ Prev Policy.

[14] Emshoff JG, Price AW (1999). Prevention and intervention strategies with children of alcoholics. Pediatrics.

[15] Grant BF (2000). Estimates of US children exposed to alcohol abuse and dependence in the family. Am J Public Health.

[16] Laslett AM, Ferris J, Dietze P, Room R (2012). Social demography of alcohol-related harm to children in Australia. Addiction.

[17] Manning V, Best DW, Faulkner N, Titherington E (2009). New estimates of the number of children living with substance misusing parents: results from UK national household surveys. BMC Public Health.

[18] McNeill A (1998). Alcohol problems in the family. A report to the European Union.

[19] Ahern K (2003). At-risk children: a demographic analysis of the children of clients attending mental health community clinics. Int J Ment Health Nurs.

[20] Bassani DG, Padoin CV, Veldhuizen S (2008). Counting children at risk. Soc Psychiatry Psychiatr Epidemiol.

[21] Maybery DJ, Reupert AE, Patrick K, Goodyear M, Crase L (2009). Prevalence of parental mental illness in Australian families. Psychol Bull.

[22] Reupert A, Maybery D (2007). Families affected by parental mental illness: a multiperspective account of issues and interventions. Am J Orthopsychiatry.

[23] Raninen J, Elgán TH, Sundin E, Ramstedt M (2015). Prevalence of children whose parents have a substance use disorder: Findings from a Swedish general population survey. Scand J Public Health.

[24] Elgán TH, Leifman H (2011). Children of substance abusing parents: a national survey on policy and practice in Swedish schools. Health Policy.

[25] Hjern A, Manhica HA. Barn som anhöriga till patienter i vården – hur många är de? Rapport 1 från projektet “Barn som anhöriga” – en kartläggning. [Children who are relatives of patients in health care - How many are there? Report 1 from the project “Children as relatives” - a survey study]. Kalmar: Nka Barn som anhöriga 2013:1. Nationellt kompetenscentrum anhöriga, Linnéuniversitetet, Chess; 2013.

[26] Ljungdahl S (2008). Barn i familjer med alkohol- och narkotikaproblem - Omfattning och analys [Children in families having alcohol and drug problems - prevalence and analysis].

[27] Wannberg H (2015). Fullständiga rättigheter! Om kommunernas stöd till barn som växer upp med missbrukande föräldrar [Full rights! On the municipalities’ support to children growing up with substance abusing parents].

[28] Määttä A, Kvillemo P. Kartläggning av insatser för barn och ungdomar i risksituationer [Survey of support activities for children and youth at risk]. In*.* Östersund, Sweden: Public Health Agency of Sweden; 2010.

[29] Andersson G (2009). Using the Internet to provide cognitive behaviour therapy. Behav Res Ther.

[30] Bennett GG, Glasgow RE (2009). The delivery of public health interventions via the internet: actualizing their potential. Annu Rev Public Health.

[31] Strecher V (2007). Internet methods for delivering behavioral and health-related interventions (eHealth). Annu Rev Clin Psychol.

[32] King R, Bambling M, Lloyd C, Gomurra R, Smith S, Reid W, Wegner K (2006). Online counselling: the motives and experiences of young people who choose the Internet instead of face to face or telephone counselling. Couns Psychother Res.

[33] Barak A, Hen L, Boniel-Nissim M, Shapira N (2008). A comprehensive review and a meta-analysis of the effectiveness of internet-based psychotherapeutic interventions. J Technol Hum Serv.

[34] Li J, Theng YL, Foo S (2014). Game-based digital interventions for depression therapy: a systematic review and meta-analysis. Cyberpsychol Behav Soc Netw.

[35] Pennant ME, Loucas CE, Whittington C, Creswell C, Fonagy P, Fuggle P, Kelvin R, Naqvi S, Stockton S, Kendall T (2015). Computerised therapies for anxiety and depression in children and young people: a systematic review and meta-analysis. Behav Res Ther.

[36] Richardson T, Stallard P, Velleman S (2010). Computerised cognitive behavioural therapy for the prevention and treatment of depression and anxiety in children and adolescents: a systematic review. Clin Child Fam Psychol Rev.

[37] Rooksby M, Elouafkaoui P, Humphris G, Clarkson J, Freeman R (2015). Internet-assisted delivery of cognitive behavioural therapy (CBT) for childhood anxiety: systematic review and meta-analysis. J Anxiety Disord.

[38] Siemer CP, Fogel J, Van Voorhees BW (2011). Telemental health and web-based applications in children and adolescents. Child Adolesc Psychiatr Clin N Am.

[39] Tönnesen H, Ståhlbrandt H, Pedersen B (2013). Web-based brief interventions for young adolescent alcohol and drug abusers – a systematic review. CLINHP.

[40] Ye X, Bapuji SB, Winters SE, Struthers A, Raynard M, Metge C, Kreindler SA, Charette CJ, Lemaire JA, Synyshyn M (2014). Effectiveness of internet-based interventions for children, youth, and young adults with anxiety and/or depression: a systematic review and meta-analysis. BMC Health Serv Res.

[41] Platt B, Pietsch K, Krick K, Oort F, Schulte-Korne G (2014). Study protocol for a randomised controlled trial of a cognitive-behavioural prevention programme for the children of parents with depression: the PRODO trial. BMC Psychiatry.

[42] Woolderink M, Smit F, van der Zanden R, Beecham J, Knapp M, Paulus A, Evers S (2010). Design of an internet-based health economic evaluation of a preventive group-intervention for children of parents with mental illness or substance use disorders. BMC Public Health.

[43] Elgán TH, Hansson H, Zetterlind U, Kartengren N, Leifman H (2012). Design of a Web-based individual coping and alcohol-intervention program (web-ICAIP) for children of parents with alcohol problems: study protocol for a randomized controlled trial. BMC Public Health.

[44] Woolderink M, Bindels JA, Evers SM, Paulus AT, van Asselt AD, van Schayck OC (2015). An online health prevention intervention for youth with addicted or mentally ill parents: experiences and perspectives of participants and providers from a randomized controlled trial. J Med Internet Res.

[45] Hodgins DC, Maticka-Tyndale E, el-Guebaly N, West M (1993). The cast-6: development of a short-form of the children of alcoholics screening test. Addict Behav.

[46] Hodgins DC, Shimp L (1995). Identifying adult children of alcoholics: methodological review and a comparison of the CAST-6 with other methods. Addiction.

[47] Schoenbach VJ, Kaplan BH, Grimson RC, Wagner EH (1982). Use of a symptom scale to study the prevalence of a depressive syndrome in young adolescents. Am J Epidemiol.

[48] Fendrich M, Weissman MM, Warner V (1990). Screening for depressive disorder in children and adolescents: validating the Center for Epidemiologic Studies Depression Scale for Children. Am J Epidemiol.

[49] Olsson G, vonKnorring AL (1997). Depression among Swedish adolescents measured by the self-rating scale Center for Epidemiology Studies-Depression Child (CES-DC). Eur Child Adolesc Psychiatry.

[50] Carver CS (1997). You want to measure coping but your protocol’s too long: consider the brief COPE. Int J Behav Med.

[51] Muhonen T, Torkelson E (2005). Kortversioner av frågeformulär inom arbets- och hälsopsykologi - om att mäta coping och optimism. Nordisk Psykologi.

[52] WHO (1997). Manual for the WHOQOL-BREF.

[53] Skevington SM, Lotfy M, O’Connell KA, Group W (2004). The World Health Organization’s WHOQOL-BREF quality of life assessment: psychometric properties and results of the international field trial. A report from the WHOQOL group. Qual Life Res.

[54] Bush K, Kivlahan DR, McDonell MB, Fihn SD, Bradley KA (1998). The AUDIT alcohol consumption questions (AUDIT-C) - An effective brief screening test for problem drinking. Arch Intern Med.

[55] Rumpf HJ, Wohlert T, Freyer-Adam J, Grothues J, Bischof G (2013). Screening questionnaires for problem drinking in adolescents: performance of AUDIT, AUDIT-C, CRAFFT and POSIT. Eur Addict Res.

[56] Andrews FM, Withey SB (1974). Developing measures of perceived life quality - Results from several national surveys. Soc Indic Res.

[57] Nagy E (2004). Barns känsla av sammanhang - En valideringsstudie av BarnKASAM i årskurserna 1–6 (ålder 7–12 år) [Children’s Sense of Coherence - A study validating SOC for children in grades 1–6 (7–12 years old)].

[58] Achenbach TM (1991). Manual for the youth self-report and 1991 profile.

[59] Achenbach TM (1991). Integrative guide to the 1991 CBCL/4-18, YSR, and TRF profiles.

[60] Saghaei M (2004). Random allocation software for parallel group randomized trials. BMC Med Res Methodol.

[61] Cohen J (1992). A power primer. Psychol Bull.

[62] Faul F, Erdfelder E, Lang AG, Buchner A (2007). G*Power 3: a flexible statistical power analysis program for the social, behavioral, and biomedical sciences. Behav Res Methods.

